# Goal Attainment Scaling in Patients With Burning Mouth Syndrome: A Real-World Clinical Study Protocol

**DOI:** 10.7759/cureus.54392

**Published:** 2024-02-18

**Authors:** Motoko Watanabe, Trang Tu, Takahiko Nagamine, Akira Toyofuku

**Affiliations:** 1 Department of Psychosomatic Dentistry, Tokyo Medical and Dental University, Tokyo, JPN; 2 Department of Basic Dental Science, University of Medicine and Pharmacy at Ho Chi Minh City, Ho Chi Minh City, VNM; 3 Department of Psychiatric Internal Medicine, Sunlight Brain Research Center, Hofu, JPN

**Keywords:** oral dysesthesia, assessment, pain management, chronic pain, burning mouth syndrome, goal attainment scaling

## Abstract

Introduction

Burning mouth syndrome (BMS) is characterized by persistent chronic burning pain. Because BMS shows various symptoms, levels of severity, and treatment outcomes, measuring recovery is difficult in this patient population. Goal attainment scaling (GAS), a flexible and responsive technique for assessing outcomes in complex interventions*,* assimilates the achievement of individual goals into a single standardized “goal attainment scale.” To our knowledge, this is the first clinical study protocol to investigate the effectiveness of adopting GAS in patients with BMS.

Methods

This study will involve two phases. In phase 1, the suitability of GAS for BMS will be examined in 30 patients. All practitioners will be trained to support patients in setting their clinical goals. In phase 2, all 155 patients with BMS will set two clinical goals emphasizing specific, measurable, achievable, realistic, and timed (SMART) goals at the initiation of psychopharmacotherapy for BMS. During the follow-up at weeks four, 8, 12, and 24, the GAS T-scores for each patient will be derived from the result of the individual goal attainment scores multiplied by goal weighting. Other clinical rating scales, including the visual analog scale (VAS), oral dysesthesia rating scale, pain catastrophizing scale, patient’s global impression of change, and clinical global improvement will be assessed simultaneously with the assessment of goal attainment. The interactions between GAS T-scores and other clinical scales or clinical characteristics, including baseline age and sex, will be analyzed, followed by a discussion on the effectiveness of adopting the GAS for BMS.

Results

The information gleaned from phase 1 will help train practitioners and develop the use of GAS for BMS. In phase 2, analyzing the GAS T-score, a quantitative assessment, will accurately reveal patient outcomes and satisfaction. The effectiveness of using the GAS and some factors contributing to patient satisfaction will be revealed by analyzing the interaction between the T-score and other clinical scales.

Conclusions

In addition to revealing the usefulness of GAS for BMS, we believe this study will prompt further investigations to clarify the factors contributing to patient satisfaction and shed light on a new treatment strategy that reinforces the previous treatments for BMS.

## Introduction

Burning mouth syndrome (BMS) is a condition characterized by persistent and chronic burning pain and unpleasant sensations in the oral region, without any corresponding abnormalities [1]. BMS presents with various symptoms, including aching, heavy, tender, and throbbing pain, and is detected mostly on the tongue, followed by the mucosa of the palatal plate, lips, and gingiva, with a wide range of severity [2] and linked to psychosocial backgrounds, including physical and psychological conditions, especially in elderly patients [1]. For symptom assessment, the visual analog scale (VAS) is useful to determine the subjective intensity of symptoms, and the oral dysesthesia rating scale (DRS) is employed to assess and quantify the severity of various oral symptoms and the degree of impairment in daily activities [3]. Pain catastrophizing scale (PCS) is generally used to assess pain catastrophizing, which is related to the chronicity of pain.

Amitriptyline, a tricyclic antidepressant, is the first-line psychopharmacological treatment for BMS in Japan, and some atypical antipsychotics, including aripiprazole, are used in pain management regimens [1]. However, the degree of their effectiveness varies for each patient, and measuring it is difficult; consequently, unmatched recognition of patients’ recovery is sometimes observed between patients and practitioners. The amount of change in the VAS and the scores of the patient’s global impression of change scale (PGIC) [4] or clinical global improvement (CGI) [5] are generally used to assess treatment outcomes. While the VAS and PGIC are self-reported questionnaires for subjective assessment, the CGI is objectively assessed by the attending doctors. These differing viewpoints may also result in discrepancies in outcome assessments.

Goal attainment scaling (GAS) was developed by Kiresuk et al. in 1968 to comprehensively assess community mental health programs [6]. It has been proposed as a patient-centered, semiquantitative measure that assimilates the achievement of individual goals into a single standardized "goal attainment score" that can be compared at the population level. It was developed in response to the growing desire to consider the goals and perspectives of patients and enable patients and healthcare providers to collaborate in setting treatment goals and measuring progress toward those goals. Each patient's problems are identified by the agreement between the physician and the patient, and treatment goals are set for each problem by using the specific, measurable, attainable, realistic, and timed (SMART) methodology. Five levels of achievement (benchmarks) are set for these goals and subsequently evaluated. Such an attempt to assess goal attainment has been used for depression and chronic pain [7-9] but has not been used in the treatment of patients with BMS. One of the essential factors for the treatment of BMS is the patient-centered approach, with a well-established relationship between patients and practitioners and shared goals of treatment. We hypothesized that adopting GAS in the treatment of BMS would result in positive outcomes. This is the first clinical study protocol to elucidate the effectiveness of the adoption of GAS in the management of patients with BMS.

## Materials and methods

Study design and patients

This prospective cohort study will include patients with BMS in Japan. Japanese outpatients diagnosed with BMS based on the International Classification of Headache Disorder third edition (ICHD-3) criteria [10] and initiating psychopharmacotherapy are deemed eligible to participate. The key exclusion criteria include patients who have been prescribed antidepressants or antipsychotics by other medical institutions and those with any neurodegenerative diseases significantly affecting cognitive functioning. This study will be conducted by adhering to the Declaration of Helsinki and the International Society for Pharmacoepidemiology (ISPE) Guidelines for Good Pharmacoepidemiology Practices (GPP). This study protocol has been registered with the University Hospital Medical Information Network (UMIN, No. 000053396), and ethical approval from the Ethical Committee of Tokyo Medical and Dental University Hospital (application no: D2023-064) will be sought. Written informed consent will be obtained from all patients before commencing the study.

**Figure 1:**
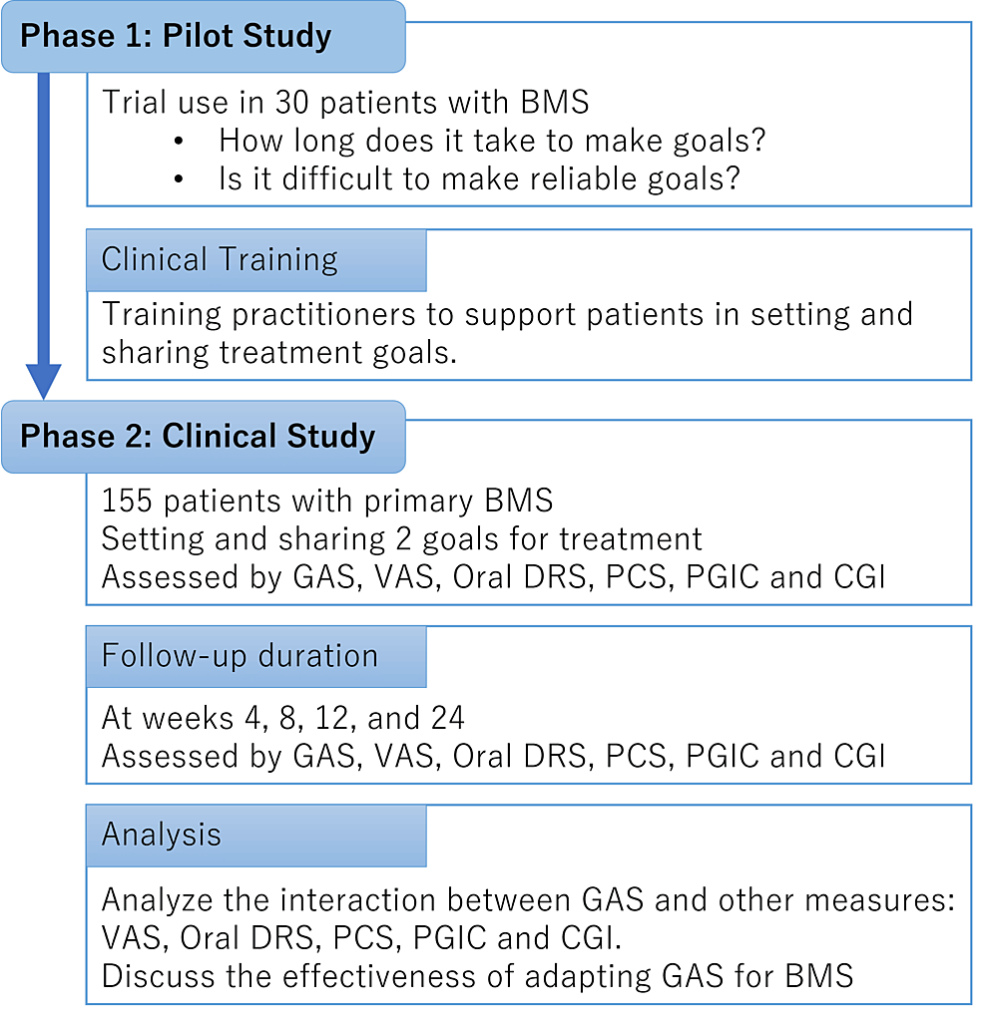
Study protocol chart In phase 1, the availability of goal attainment scaling (GAS) for burning mouth syndrome (BMS) will be examined in 30 patients with BMS. All practitioners will be trained to support patients in setting their clinical goals. In phase 2, all 155 patients with BMS will set two clinical goals, focusing on setting specific, measurable, achievable, realistic, and timed (SMART) goals at the initiation of psychopharmacotherapy for BMS and will be followed up at weeks four, eight, 12, and 24 to assess progress according to the GAS protocol. The GAS T-score will be calculated, and its interaction with other clinical characteristics and clinical scales, including visual analog scale (VAS), oral dysesthesia rating scale (Oral DRS), pain catastrophizing scale (PCS), patients' global impression of change scale (PGIC), and clinical global improvement (CGI), will be assessed at the same time as goal attainment

In phase 1, as a pilot study, the availability of GAS for BMS, including the possibility and time to set reliable goals in a clinical situation, will be examined in 30 patients with BMS (Figure 1).

All practitioners should be trained to support patients in achieving their clinical goals by considering their personal conditions and availability. In phase 2, 155 BMS outpatients will be involved. The required sample size has been estimated based on a threshold response rate of 50% [11], an expected response rate of 60%, 80% power, and an alpha value of 0.1 (one-sided) by using the binomial test. Assuming 2% of ineligible patients, the target sample size has been determined to be at least 155 patients. All patients will set two clinical goals at the initiation of psychopharmacotherapy and assess their attainment during BMS treatment, as described below. Previous scaling tools will also be provided at the time of setting goals and assessing their attainment. To evaluate the usefulness of the GAS, the relationship between the GAS and other assessment tools and satisfaction with the GAS in both patients and practitioners will be analyzed.

Goal setting and goal attainment scaling

Patients will build their clinical goals with an emphasis on setting SMART goals by trained practitioners. Using the SMART framework, practitioners should consider the attainability of goals, the use of observable objectives and benchmarks, equidistance of scaling, the expected level of difficulty in achieving goals, goal differentiation, and the overall quality of goal statements. Goals are ranked according to their importance to the patient (1 = least important and 2 = most important) and achievability (1 = easy, 2 = average difficulty, and 3 = difficult). When more than two goals are given, they will be selected for the assessment of attainment according to their importance to the patient and reliability over negotiations between the patient and practitioner. Examples of the goal statements are listed in Table 1.

**Table 1:** Examples of goal statements with the ranking of importance and achievability and description of their outcome values VAS: visual analog scale

Goals	Goal weight	Achievability	Outcome value					Decision
			Most unfavorable, baseline performance (-2)	Less than expected (-1)	Expected level (0)	More than expected success (+1)	Best anticipated treatment (+2)	
Chewing gum	1	2	In a month, the number of days of chewing gum did not change	In a month, the number of days of chewing gum reduced to 2/3rds	In a month, the number of days of chewing gum reduced to half.	In a month, the number of days of chewing gum reduced to 1/3rds	In a month, no chewing gum to reduce pain	Not selected
Exercise	1	1	In 3 months, could not go out for a walk at all	In 3 months, 10 minutes of walking a day every week	In 3 months, 10 minutes of walking in 3 days every week	In 3 months, 10 minutes of walking in 5 days every week.	In 3 months, 10 minutes of walking every day	Selected
VAS	2	2	In 6 months, VAS scores did not reduce or increase	In 6 months, VAS scores reduced to 2/3rds	In 6 months, VAS scores reduced to half	In 6 months, VAS scores reduced to 1/3rds	In 6 months, VAS scores reduced to 1/4ths	Selected

Patients and clinicians will meet at baseline to determine personal treatment goals, which will be followed up at weeks four, eight, 12, and 24 to assess progress. Each goal has its own written goal statements with levels of possible achievement set on a predefined 5-point rating scale (-2: 0% goal achievement, most unfavorable and baseline performance; -1: less than expected; 0: 100% goal achievement and expected level; +1: more than expected success; +2: 200% goal achievement, and the best anticipated treatment), according to the GAS protocol [6].

The GAS T-score for each patient was derived from the product of individual goal attainment scores multiplied by goal weighting using the following standard formula, where w_i_ is the weight assigned to the i^th^ goal (if equal weights, w_i_ = 1) and x_i_ is the number of outcome values (between -2 and +2).

$T-score= 50+\frac{10\sum_{i= 1}^{k}w_{i}x_{i}}{\sqrt{0.7\sum_{i= 1}^{k}w_{i}^{2}+0.3(\sum_{i= 1}^{k}w_{i})^{2}}}$

The GAS T-scores are standardized such that the average GAS T-score is 50, and the standard deviation is 10, assuming that the goals are set without bias. Thus, a score of 50 indicates that the goal is achieved on average as expected, a score below 50 indicates that the goal is achieved at a less-than-expected level, and a score above 50 indicates that the goal is achieved at a more-than-expected level. 

Other clinical assessments for outcomes

Outpatient psychopharmacotherapy will be initiated by psychosomatic dentists, and patients will be followed up for 24 weeks, with visits planned at baseline and at weeks four, eight, 12, and 24. The primary outcome measure for the main study is GAS. Other clinical rating scales, including the VAS, Oral DRS, PCS, PGIC, and CGI, will be assessed as secondary outcomes. Permission to use the Japanese version of the PCS was obtained from all the authors. At the end of the study (week 24), the patients and practitioners will be asked to rate the usefulness of the GAS approach in establishing treatment goals, monitoring progress, and helping achieve successful treatment outcomes. Each aspect of satisfaction is assessed on a 7-point scale (1 = least useful and 7 = most useful).

Data analysis

Analyses will be conducted using the full analysis set (FAS), which will include all eligible patients who have undergone psychopharmacotherapy and completed a baseline visit and one or more follow-up visits. Changes from baseline in GAS T-scores will be analyzed using a two-sided test, and changes from baseline in traditional clinical rating scales will be analyzed using a restricted maximum likelihood (REML)-based mixed model for repeated measures (MMRM) with baseline age, sex, visit, baseline score, and baseline score-by-visit interactions included as fixed effects.

To assess convergent validity, Spearman’s rank correlations between changes in GAS T-scores from baseline and traditional clinical measures will be evaluated at baseline and weeks four, eight, 12, and 24. Test-retest reliability for the GAS will be assessed by the intraclass correlation coefficient (ICC) by using data from weeks eight and 12 in those who do not report changes in PGIC (i.e., those with the same PGIC scores at weeks eight and 12).

## Results

Anticipated results

**Figure 2:**
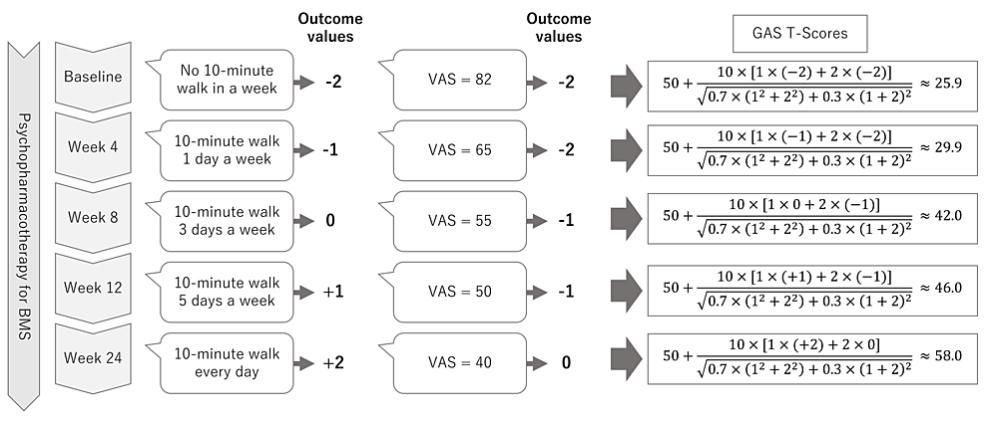
Examples of outcome values and derivations of goal attainment scaling (GAS) T-scores for each goal Outcome values are assessed for each goal at baseline, with initiation set as the baseline. GAS T-scores were derived from the goal-weighted goal and outcome value according to the standard formula. In this case with burning mouth syndrome (BMS), the goals are achieved as much as or more than expected by week 24

The information gleaned in phase 1 will help train practitioners and develop the use of GAS for BMS. When setting goals that are reliable and achievable, attainment would be possible despite the severity of BMS symptoms or other psychological backgrounds in phase 2. Outcome values for each goal will be scored, followed by the derivation of GAS T-scores at baseline and weeks four, eight, 12, and 24, as shown in Figure 2.

Analyzing the T-score, which is a quantitative assessment, will accurately reveal outcomes and patient satisfaction, although patients have different treatments, severities, sensations, and outcomes. The effectiveness of adapting the GAS for BMS and the factors contributing to patient satisfaction will be revealed by analyzing the interaction between the T-score and other clinical scales.

## Discussion

This is the first study protocol to adapt the GAS for BMS, a form of chronic oral pain characterized by burning and various other types of pain with a wide range of severities, in the absence of a corresponding organic cause. Pharmacotherapy with antidepressants is effective for BMS [1]. However, due to different patient conditions and detailed treatment strategies, patient satisfaction with treatment varies and is not always high, resulting in patients undergoing a variety of tests, incurring high treatment costs, and developing somatization [12]. The evaluation of pharmacotherapy using pain indices does not necessarily correlate with recovery in patients with BMS. Medication is an active process that involves complex decision-making and opportunities to work through decisional conflicts [13]. Recovery is the process by which people can live, work, learn, and participate in their communities. For some individuals, recovery is the ability to lead full and productive lives despite disabilities. While the GAS is useful as an indicator of personal recovery, it is also expected to be a treatment tool that may have a synergistic effect on previous treatment strategies through shared decision-making (SDM) [7,8,14]. Patients would be more motivated to achieve clinical goals for recovery by being involved in setting and attaining goals that are reliably set for each step.

The results of this study are expected to answer the following questions. Firstly, whether the effectiveness of psychopharmacotherapy in patients with BMS can be evaluated using both traditional pain measures and new recovery measures. The GAS has been reported to be an effective evaluation tool for treatment with medications, especially in studies with small sample sizes [15]. Because the prevalence of BMS has been reported to be 0.7-3.7% [1], the sample size of investigations on BMS tends to be small. Therefore, adapting the GAS to study the effectiveness of psychopharmacotherapy for BMS is appropriate and will reveal more accurate and real-world results. Secondly, whether the relationship between traditional pain measures and new recovery measures can be assessed. The results will reveal whether there is a correlation or divergence between pain improvement and personal recovery. Third, by assessing treatment satisfaction, the process by which practitioners and patients determine treatment goals can be examined for their potential to produce therapeutic effects. This might help understand patients’ recognition of recovery and establish better doctor-patient relationships [1].

Through this study, clinicians could learn the essence of shared decision-making and help patients make appropriate decisions (goals). Setting goals is critical and the interaction between patients and practitioners in sharing clinical goals is an essential element of patient satisfaction. GAS is a method of promoting patient recovery based on SDM, in which healthcare professionals and patients work together to set treatment goals, and has been shown to be effective in the treatment of depression [7,8,16]. Although this is the first time GAS has been applied to chronic pain associated with dental psychosomatic disorders, it is expected to help patients recover.

This study has several limitations. GAS might not simply reflect the improvement of BMS, as patients with BMS often have comorbid somatic symptoms, including headache and chronic pain in other parts of the body [17], which interact in a complicated way with BMS. However, the strength of this study is that it attempts to investigate real-world clinical situations for BMS with a comprehensive outlook.

## Conclusions

The key to this research plan lies in determining the goals of patients with BMS by using SDM. Adapting the GAS to the treatment for BMS will motivate patients and help achieve better prognosis by establishing better doctor-patient relationships. In addition to revealing the usefulness of GAS for BMS, this study protocol will lead to further investigations to clarify the factors contributing to patient satisfaction and shed light on other treatment strategies that reinforce previous treatments for BMS. Moreover, the use of GAS is expected to expand to other oral psychosomatic disorders that present with chronic uncomfortable sensations, following further studies in the future.
